# Solvothermal synthesis, growth mechanism, and photoluminescence property of sub-micrometer PbS anisotropic structures

**DOI:** 10.1186/1556-276X-7-668

**Published:** 2012-12-06

**Authors:** Yali Cao, Pengfei Hu, Dianzeng Jia

**Affiliations:** 1 Key Laboratory of Advanced Functional Materials of Autonomous Region, Institute of Applied Chemistry, Xinjiang University, Urumqi, Xinjiang, 830046, China; 2 Key Laboratory of Clean Energy Material and Technology of Ministry of Education, Institute of Applied Chemistry, Xinjiang University, Urumqi, Xinjiang, 830046, China; 3 Laboratory for Microstructures, Shanghai University, Shanghai, 200444, China

**Keywords:** Nanostructures, Semiconductors, Chemical synthesis, Crystal growth, Photoluminescence

## Abstract

The sub-micrometer PbS with anisotropic microstructures including fishbone-like dendrites, multipods, cubes, corallines, and hopper cubes were successfully prepared by the solvothermal process. Different morphologies can be obtained by adjusting the reaction temperatures or using the nontoxic controlled reagents which can tune the relative growth rate in the <100> direction and the <111> direction of PbS nuclei. Based on the viewpoint of crystallography about face-centered cubic crystal structure, the possible formation mechanism for sub-micrometer anisotropic structures has been discussed. The difference between the enhanced growth rates on the {100} and {111} planes induced the change of ratio between the growth rates in the <100> and <111> directions, which resulted in the formation of the different PbS anisotropic microstructures. The PbS anisotropic structures exhibited the different visible emission with a peak in the red regions mainly attributed to the variation of shape, size, and the trap state of as-obtained PbS.

## Background

With a near-infrared direct narrow bandgap (0.41 eV at 300 K) and a large exciton Bohr radius (18 nm), lead sulfide (PbS) is an important π-π semiconductor material [1,2]. The sub-micrometer structures of PbS, which display unique physical properties originating from the intrinsic nature of the bulk materials [3,4], have been promising for optoelectronic devices such as Pb^2+^ ion-selective sensors [5,6], near-IR communication and switches [7,8], flame monitors [9,10], photonic and optical switching devices [11,12], etc. It is well known that the intrinsic properties of materials, including the micrometer- and nanometer-sized crystals, rely sensitively on their crystallinity, morphologies, and surfaces [13-15]. Therefore, it is essential to design a reasonable synthetic process for the synthesis of micrometer- or nanometer-sized PbS crystals with desired morphology [16,17].

Over the past few years, researches aiming at the possibility to control the microstructure of the crystals have been developed [18-20]. Some special structures, such as wire-, rectangle-, rod-, star-, and cube-like crystals have been demonstrated [21,22]. Various surfactants were used to change the microenvironment or affect the growth process for solution synthesis [23,24] and then inorganic nanomaterials with different morphologies were obtained [25,26]. For the synthesis, the general strategy is to handle specific inducing agent which can selectively combine with specific crystal facets [22,27]. Thus, the growth rates on different facets can be enhanced or weakened and consequently produce final crystals with different morphologies [28,29]. Recently, PbS nanocrystals with peculiar anisotropic morphology have aroused the interest, and some anisotropic structures were synthesized through a solution route [21,30]. This special anisotropic structure may lead to the different physical and chemical characteristics from the bulk or one-dimensional structure. It inspired us to seek suitable reagent for the synthesis of anisotropic PbS microstructures in high yield.

Here, the sub-micrometer-sized PbS anisotropic structures with morphology of fishbone-like dendrites, multipods, cubes, corallines, and hoppers were successfully synthesized by adjusting the reaction temperature or employing the nontoxic reagent to vary the relative growth rate on different crystalline facets of PbS nucleating seeds under the solvothermal conditions. The structure evolution and formation mechanism of PbS anisotropic microstructures have been discussed. The photoluminescence property was also investigated.

## Methods

In this paper, all the reagents were of analytical grade and were used without further purification. The synthesis of sample was carried out through the solvothermal process. In a typical experiment, 20 mL of 0.05 mol/L lead acetate solution was added into stirred 20 mL of 0.05 mol/L 1-pyrrolidine dithiocarboxylic acid ammonium salt solution in methanol. A thick suspension of white precipitate appeared immediately. Subsequently, the above dispersion was transferred into a 50-mL Teflon-lined stainless steel autoclave, sealed, and maintained at 165°C or 180°C for 12 h, respectively. As to the solvothermal route with surfactant sodium dodecylbenzenesulfonate (SDBS), different masses of SDBS were added into the above mother mixture solution, respectively, and maintained at 180°C for 12 h. Then the autoclaves were cooled to room temperature naturally. The collected products were then washed with distilled water and methanol several times and dried under vacuum at 60°C for 5 h before further characterization. The processes with other compounds cetytrimethylammonium bromide (CTAB), ethylenediamine tetraacetic acid (EDTA), sodium dodecyl sulfate (SDS), cellulose triacetate (CTA), and polyethylene glycol 400 (PEG-400) were similar to that of SDBS.

Powder X-ray diffraction (XRD), transmission electron microscopy (TEM), and scanning electron microscopy (SEM) were applied to characterize the crystallographic phase, size, and morphology evolution of the products. Powder XRD measurement was performed with a MXP18AHF X-ray diffractometer (MAC Science Co. Ltd., Tokyo, Japan) using CuK*α* radiation (*λ* = 0.154056 nm). TEM images were collected using HITACHI H-600 transmission electron microscope (Chiyoda, Tokyo, Japan) at the operating voltage of 75 kV. SEM images were conducted at 15 keV with a LEO 1430VP scanning electron microscope (Carl Zeiss AG, Oberkochen, Germany).

## Results and discussion

**Figure 1:**
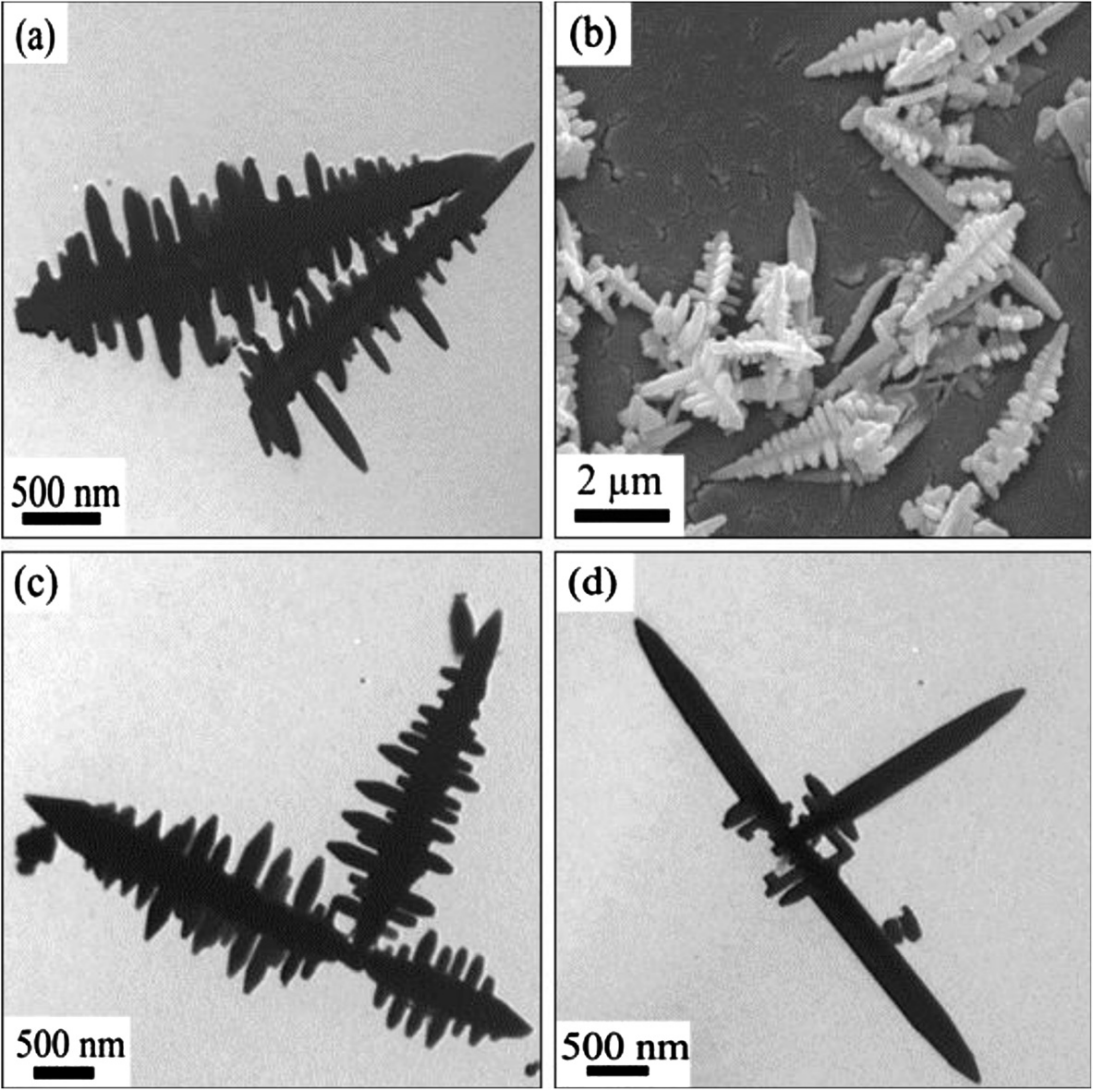
**TEM and SEM images of the fishbone-shaped PbS crystals.** Obtained via solvothermal route without surfactants at 165°C for 12 h. (**a**) Dendrites, (**b**) three-dimensional dendritic structures, (**c**) hierarchical dendritic structures, and (**d**) incomplete dendrites.

Figure 1 shows TEM and SEM images of PbS prepared at 165°C without surfactants. It is shown that the products are mainly fishbone-like dendritic structures. Each dendrite contains a main ‘trunk’ with small lateral ‘branches’ growing perpendicularly to the major trunk (Figure 1a). The typical SEM of individual dendrite clearly reveals a view of its three-dimensional dendritic structure with four rows of branches. It can be also seen that the separation angle between the adjacent rows is 90°. Each row is composed of some branches. These branches are parallel to each other orderly and perpendicular to the trunk (Figure 1b). The main trunks are similar to these branches in the shape. Each of them has a sharp cusp like a sword. The main trunk has the length of about 1.0 to 3.5 μm and the diameter of 100 nm. The branches are 50 to 500 nm in length and 50 to 80 nm in diameter. In addition, the further hierarchical dendritic structures constructed by fishbone-like dendrites formed in the process (Figure 1c). Some incomplete dendrites were also observed in the sample. The growth of their branches aborted, while the trunks have been full-grown (Figure 1d).

**Figure 2:**
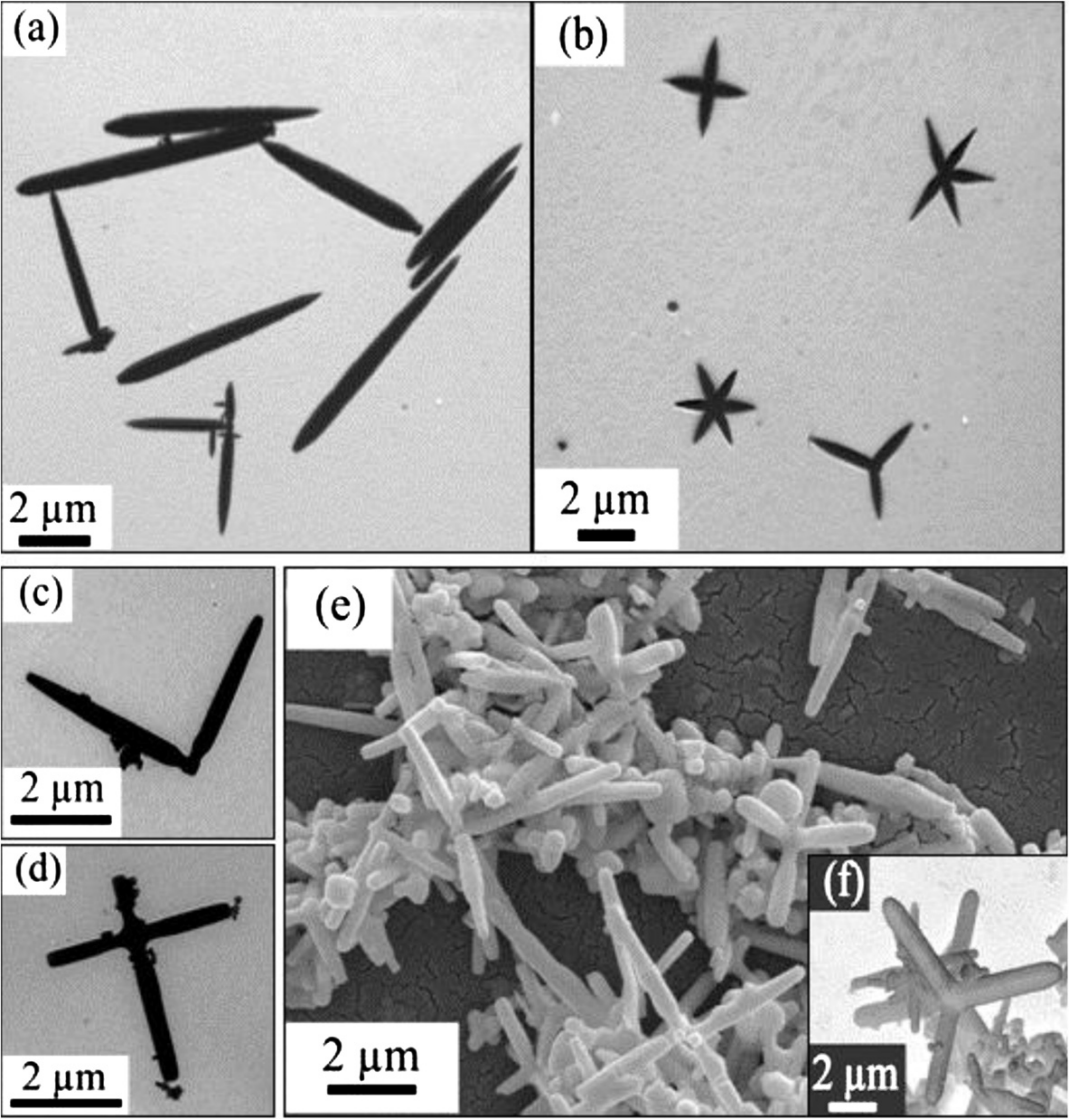
**TEM and SEM images of the pod-shaped PbS microstructures.** Obtained via solvothermal process without surfactant at 180°C for 12 h. (**a**) Monopods, (**b**, **c**, and **d**) cross-shaped structures, and (**e** and **f**) three-dimensional cross-shaped structures.

When the growth of the materials was carried out at 180°C, dramatic changes occurred to the structures of products. Figure 2 shows the SEM and TEM images, which distinctly reveal the sizes and morphologies of the as-obtained products. It is seen that the product prepared at 180°C is composed of a majority of pod-based crystals without the lateral smaller ‘sticks’. It has structures ranging from monopodal to hexapodal shapes. Besides a number of monopods in Figure 2a, cross-shaped structures are the main shape which consisted of two to six pods (Figure 2b,c,d). Typical SEM images are present in Figure 2e. Three-dimensional PbS crystals composed of six cross-shaped pods were vividly displayed. From Figure 2f, it can be seen that the separation angle between the two adjacent crossed pods is 90°. It is estimated to be 1 to 3 μm in length and 200 to 500 nm in diameter for each pod. For a certain unit, the diameter of each pod is nearly identical. In addition, a few crucifix-shaped tetrapods can be found in this sample (Figure 2d).

**Figure 3:**
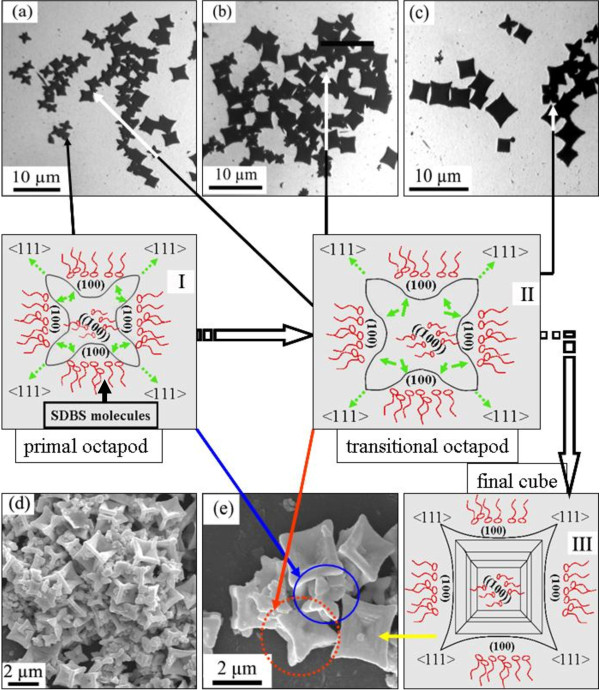
**TEM and SEM images of the samples.** Samples synthesized with 3.6 × 10^−3^ M SDBS at the reaction temperature of 180°C under different duration times of **(a)** 3 h, **(b)** 6 h, and **(c, d,** and **e)** 12 h. (I to III) Schematic illustration of the evolution process of the PbS hopper cubic structures: from (I) primal octapod (corresponding to the object marked by blue circle in image e) to (II) transitional octapod (corresponding to the object marked by red circle in image e) to (III) final cube (corresponding to the object marked by yellow arrow in image **e**).

To explore the influence of the surfactant SDBS to the morphology of PbS microcrystals, the following experiments were carried out: Firstly, we designed several solvothermal processes in the presence of different concentrations of SDBS (its CMC is 1.2 × 10^−3^ M), respectively, at the reaction temperature of 180°C and holding time for 12 h. TEM and SEM images of the products indicated that the surfactant SDBS can induce new structures (Figure 3) [Additional file 1: Figure S1]. It is worth noting that all products prepared with different concentrations of SDBS contain the novel cubic structure. It is clearly shown in Figure 3d,e that each cube has a perfect cubic frame with six pyramid-shaped hoppers negative toward the center on the {100} facets and eight elongated horns along the <111> directions. In addition, some hoppers exposed visual step-like faces, i.e., a family of {100} facets. The experimental data showed that the reaction with 3.6 × 10^−3^ M SDBS can produce this structure with high yield. Reactions with 0.8 × 10^−3^ M or 1.2 × 10^−3^ M or 7.2 × 10^−3^ M SDBS created farraginous structures, cubes, truncated cubes, particles, or shuttle-like crystals. Subsequently, the several reactions with 3.6 × 10^−3^ M SDBS were developed for different duration times to study the evolution of PbS microstructure with time. Figure 3a,b,c presented the evolution of the structure from multipods to fat pods to final cubic frames along the duration time, and they indicated that the number of cubes with the hopper increased when the time was prolonged. Further investigation discovered that the shape evolution casts anchor at about 12 h. The exhaustion of as-obtained PbS monomer is responsible for the evolution abortion of some pods.

Indeed, the route in this work was a thermal decomposition of uni-precursor {[Pb(C_5_H_8_NS_2_)_2_]_2_} through a solvothermal reaction. When the lead acetate (Pb(AC)_2_) solution was added into 1-pyrrolidine dithiocarboxylic acid ammonium salt (C_5_H_12_N_2_S_2_) solution, the thick suspension of white precipitate came into being. We separately prepared the precursor with identical solutions and manipulation. The TG-DTA and element analyses of precursor were described in details in the Additional file 1.

The formation of PbS sub-micrometer structures involves nucleation and subsequent growth processes of nuclei. The rock salt structure of the PbS seed crystallite formed during the nucleation process is the initial factor responsible for the final shape of the PbS structures. It is generally considered that these PbS seeds are tetradecahedrons (truncated cube), exposing six {100} facets and eight {111} facets. According to the literature, the final shape of a PbS nanocrystal was mainly determined by the ratio of the growth rate in the <100> direction to that in the <111> direction. From the viewpoint of crystallography, the {111} facets of a rock salt structure intrinsically have a higher surface energy than that of the {100} facets, and the growth kinetic energy barrier of the crystal facet is inversely proportional to the surface energy. This suggests that the growth rate along the <111> direction is higher than that of the <100> direction. However, the growth environment can influence the formation of anisotropic structures, dendrites, multipods, and cube with hoppers. Lifshitz mentioned that the main environmental parameters are as follows: (a) the stabilizing surfactants which can enhance or block the growth of specific facets, (b) the reaction temperature and reaction duration which determined the choice of thermodynamic or kinetic growth, and (c) a large concentration of the precursors to enable a fast kinetic growth and Ostwald ripening effect [21]. In a word, the final structure of a NaCl-type PbS crystal can be controlled by modulation of the ratio (R) between the growth rates in the <100> and <111> directions. As illustrated by Wang [31], the R for the formation of octahedra is 1.73, and that for the perfect cubes shape is 0.58.

In this work, when the reaction temperature was 165°C and 180°C, the growth rates were fast. This led to a preferential kinetic growth of branches. The difference between the enhanced growth rates on the {100} and {111} planes induced the ratio R to have a value of more than 1.73, which resulted in the formation of the dendrites as shown in Figure 1a,c. As to the disappearance of the lateral smaller sticks at 180°C, the higher temperature may be an important factor. The relatively higher growth rate in <100> direction than that in <111> direction leads to the formation of the pods. Further exploration to this behavior is progressing in our laboratory.

In some corresponding reports, the synthesis of special PbS nanostructures was usually achieved with the stabilizing surfactants. These surfactants are mostly amine compounds, such as ethylene diamine (en). Ma et al. proposed that the amine molecules stabilize the {100} surfaces and, thus, enhance the natural habit of the rock salt structure to react faster along the <111> direction [30]. In our work, we found that the surfactant SDBS can also play the same role. When it was added to the reaction system, SDBS can rapidly increase the growth kinetic coefficient between the pods. However, the {100} surfaces of the original tetradecahedron were still blocked by the surfactant SDBS molecules, and the rate R was reduced to 0.58. The evolution of microstructures from octapod to final cube was schematically illustrated in Figure 3. The primal octapod (I) had a central cube with eight centrosymmetric pods pullulating along the apex. It was clearly shown in the SEM photograph (marked with the blue continuous circle in Figure 3e). These octapods gradually grew into intermediate shape (II) and ripened to final cubes when the reaction proceeded. Some transitional shapes evolved between the octapods, and the ripe cubes were mostly generated in the products obtained at 3 and 6 h (Figure 3a,b). They were also confirmed by the SEM micrograph (marked with the red dot circle in Figure 3e). These structures formed by growing the space between the pods without further growth of the central cube, and their pods kept on stretching to the eight apexes of the central cube. Thus, it was doubtless that the pods gradually gathered flesh and pullulated to become developmental fat octapods. The distance from the neighboring pods is about 2 μm (Figure 3). The continuative growth of the aforementioned mode resulted in the formation of final sculpturesque cubes (III) by filling the space between the arms without further growth of the {100} facets of the central cube. These cubes are about 2.5 × 2.5 μm in size.

**Figure 4:**
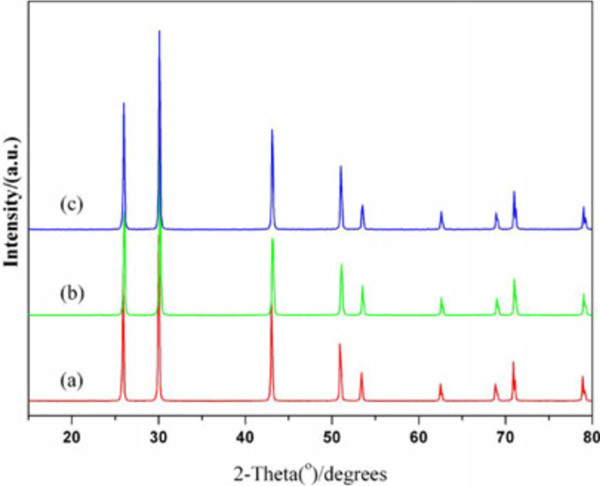
**X-ray powder diffraction patterns of microstructures.** (**a**) Fishbone-shaped, (**b**) pod-shaped, and (**c**) hopper cubic microstructures.

The crystallinity of the as-prepared dendrites, multipods, and hopper cubes are clearly illustrated by their distinct XRD patterns shown in Figure 4. All the peaks can be indexed as face-centered cubic structure with the Fm3m space group (JCPDS no. 05–592). The high intensity of the diffraction patterns suggests that the samples have been well crystallized. The XRD pattern of cubic crystal gives an average lattice parameter of 0.5927 nm (calculated from (200) and (111) peaks in Figure 4c), which is in good agreement with the JCPDS no. 05–592 database (*a* = 0.5936 nm).

**Figure 5:**
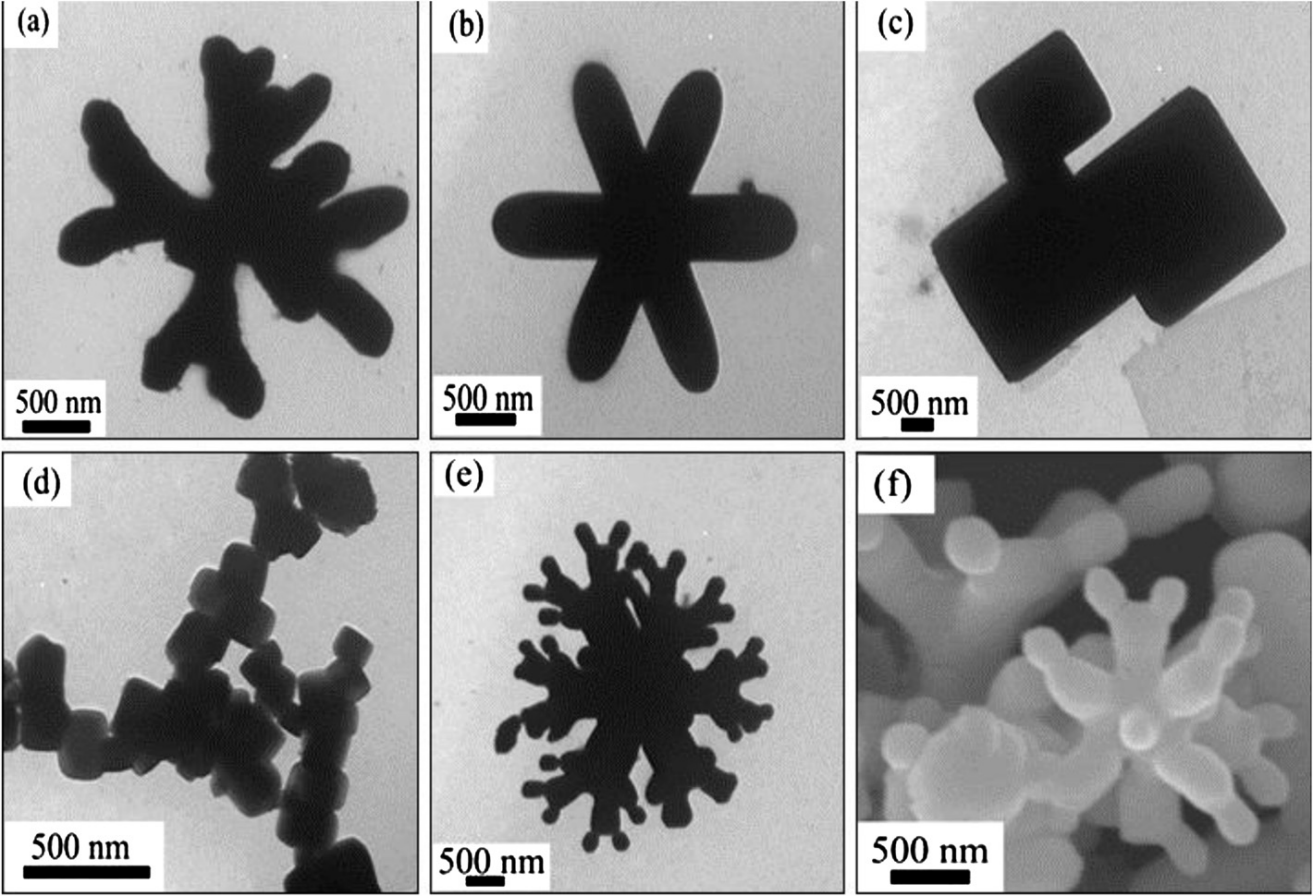
**TEM and SEM images of the PbS microstructures.** Obtained with different surfactants via solvothermal processes at 180°C for 12 h, respectively. **(a)** CTAB, **(b)** SDS, **(c)** EDTA, **(d)** CTA, and **(e** and **f)** PEG-400.

In addition, the present works indicated that the compounds of CTAB, EDTA, SDS, CTA, and PEG-400 can also modulate the relative growth rate on the different surfaces of NaCl-type PbS crystals. Some novel anisotropic microstructures have been created through the solvothermal processes with their assistant. When CTAB was added into the system, dendritic PbS microcrystals were obtained (Figure 5a). The cross-shaped PbS with plump pods responded to surfactant SDS (Figure 5b). The present work showed that the EDTA can decrease the R between the growth rates in the <100> and <111> directions. The full cubic structure would come into being with them (Figure 5c). As to CTA, it can diminish the size of the products. The nanocubes or nanobars synthesized with CTA are about 70 to 150 nm in width (Figure 5d). The vivid coralloid structures were fabricated with the nonionic surfactant PEG-400 (Figure 5e). Figure 5f clearly revealed that the buds of the coralline were the PbS nanocubes or nanobars grafted on the PbS stakes. Furthermore, under the same experimental condition (the reaction temperature, duration time, and solvents are equal to the aforementioned process without surfactants), the molar ratio of Pb^2+^/S^2−^ source (1:1) were replaced by 2:1 and 3:1. The results showed that the molar ratio of Pb^2+^/S^2−^ weakly affects the final morphologies of PbS crystals in the present case.

**Figure 6:**
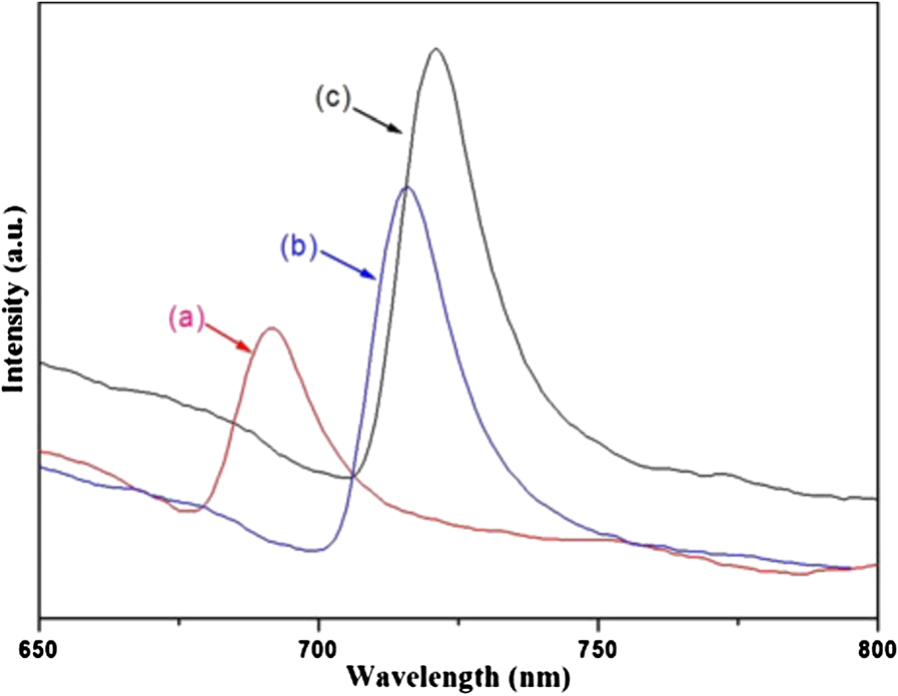
**Photoluminescence spectrum of PbS microstructures obtained with different processes.** (**a**) Without surfactant at 165°C for 12 h (T165), (**b**) without surfactant at 180°C for 12 h (T180), and (**c**) with 3.6 × 10^−3^ M SDBS at 180°C for 12 h (T180_SDBS_).

Figure 6 shows the room-temperature photoluminescence (PL) spectra of the PbS microstructures obtained by different processes. It is harvested that these PL spectra show a clear shape- and size-dependent features. With the excitation wavelength of 485 nm, the emission maximums of the PL spectra of T165, T180, and T180_SDBS_ are around 692, 716, and 721 nm with a near equal narrow FWFM of approximately 20 nm, while the intensities of T180 and T180_SDBS_ are stronger than T165. A great deal of investments suggested that the PL performances of nanoparticles are generally impacted by the shape, dimensions, size, size distribution, or density of defects. In the present work, the sharp optical spectra in three PL imply the narrow size distribution and blueshift compared to the bulk counterpart (NIR region) suggesting the strong degree of confinement. The samples T180 and T180_SDBS_ should hold better crystallinity than T165 and possess lower density of defects. The opportunities of nonradiative recombination of photoinduced electron–hole pairs will be depressed, so the intensities of their PL spectra are higher than T165. It is considered that the differences of emission peaks are mainly attributed to the variation of shape, size, and the trap state of as-obtained PbS. The lower wavelengths of T165 and T180 implied the stronger confinement than T180_SDBS_, which can be understood from the fact that T165 and T180 are composed of one-dimensional units with smaller sizes simultaneously, while the T180_SDBS_ has no one-dimensional structures.

## Conclusions

In conclusion, the solvothermal route has been successfully developed to prepare sub-micrometer PbS anisotropic structures with different morphology. The solvothermal synthesis of PbS anisotropic structures including fishbone-like dendrites, multipods, cubes, corallines, and hoppers has been facilely applied by adjusting the reaction temperature or using the different nontoxic reagents. The results indicated that the modulation of reaction condition with different surfactants, which can adjust the ratio between the growth rates in the <100> and <111> directions, is the key for the fabrication of the NaCl-type PbS microstructures.

## Competing interests

The authors declare that they have no competing interests.

## Authors’ contributions

YC and PH carried out the preparation of the samples, analysis of the data, the proposition of the mechanism and wrote the manuscript. DJ participated in the design of the experiments and performed the photoluminescence analysis. All authors read and approved the final manuscript.

## Supplementary Material

Additional file 1 **The structure synthesized with different SDBS concentrations was shown in Figure S1.** The TG-DTA and element analyses of precursor were described in **Figure S2** and **Table S1**. Click here for file

## References

[B1] Machol JL Wise FM Patel RC Tanner DB Vibronic quantum beats in PbS microcrystallites Phys Rev B 1993 48 2819 2822 10.1103/PhysRevB.48.2819 10008690

[B2] Kane RS Cohen RE Silbey R Theoretical study of the electronic structure of PbS nanoclusters J Phys Chem 1996 100 7928 7932 10.1021/jp952869n

[B3] Henglein A Small-particle research: physicochemical properties of extremely small colloidal metal and semiconductor particles Chem Rev 1989 89 1861 1873 10.1021/cr00098a010

[B4] Levina L Sukhovatkin W Musikhin S Cauchi S Nisman R Bazett-Jones DP Sargent EH Efficient infrared-emitting PbS quantum dots grown on DNA and stable in aqueous solution and blood plasma Adv Mater 2005 17 1854 1857 10.1002/adma.200401197

[B5] Wang Y Nonlinear optical properties of nanometer-sized semiconductor clusters Acc Chem Res 1991 24 133 139 10.1021/ar00005a002

[B6] Patel AA Wu F Zhang JZ Torres-Martinez CL Mehra RK Yang Y Risbud SH Synthesis, optical spectroscopy and ultrafast electron dynamics of PbS nanoparticles with different surface capping J Phys Chem B 2000 104 11598 11605 10.1021/jp000639p

[B7] Sargent EH Infrared quantum dots Adv Mater 2005 17 515 522 10.1002/adma.200401552

[B8] Ge JP Wang J Zhang HX Wang X Peng Q Li YD Orthogonal PbS nanowire arrays and networks and their Raman scattering behavior Chem Eur J 2005 11 1889 1894 10.1002/chem.200400633 15685583

[B9] Stchur P Cleveland D Zhou J Michel RG A review of recent applications of near infrared spectroscopy, and of the characteristics of a novel PbS CCD array-based near-infrared apectrometer Appl Spectrosc Rev 2002 37 383 428 10.1081/ASR-120016293

[B10] Chahadih A Hamzaoui H Bernard R Bois L Beclin F Cristini O Capoen B Bouazaoui M Continuous laser direct-writing of PbS nanoparticles inside transparent silica monoliths J Nanopart Res 2011 13 6507 6515 10.1007/s11051-011-0554-1 PMC3224756 21970510

[B11] Ellingson RJ Beard MC Johnson JC Yu P Micic OI Nozik AJ Shabaev A Efros AL Highly efficient multiple exciton generation in colloidal PbSe and PbS quantum dots Nano Lett 2005 5 865 871 10.1021/nl0502672 15884885

[B12] Mcdonald SA Konstantatos G Zhang S Cyr PW Klem EJD Levina L Sargent EH Solution-processed PbS quantum dot infrared photodetectors and photovoltaics Nat Mater 2005 4 138 143 10.1038/nmat1299 15640806

[B13] Burda C Chen XB Narayanan R EI-Sayed MA Chemistry and properties of nanocrystals of different shapes Chem Rev 2005 105 1025 1102 10.1021/cr030063a 15826010

[B14] Acharya S Gautam UK Sasaki T Bando Y Golan Y Ariga K Ultra narrow PbS nanorods with intense fluorescence J Am Chem Soc 2008 130 4594 4595 10.1021/ja711064b 18338893

[B15] Zhang Y Dai QQ Li XB Cui QZ Gu ZY Zou B Wang YD Yu WW Formation of PbSe/CdSe core/shell nanocrystals for stable near-infrared high photoluminescence emission Nanoscale Research Lett 2010 5 1279 1283 10.1007/s11671-010-9637-7 PMC2897039 20676204

[B16] Wang CW Liu HG Bai XT Xue QB Chen X Lee YI Hao JC Jiang JZ Triangular PbS nano-pyramids, square nanoplates, and nanorods formed at the air/water interface Cryst Grow Des 2008 8 2660 2664 10.1021/cg070398b

[B17] Wang SH Yang SH Preparation and characterization of oriented PbS crystalline nanorods in polymer films Langmuir 2000 16 389 397 10.1021/la990780t

[B18] Xiong SL Xi BJ Xu DC Wang CM Feng XM Zhou HY Qian YT l-cysteine-assisted tunable synthesis of PbS of various morphologies J Phys Chem C 2007 111 16761 16767 10.1021/jp075096z

[B19] Ni YH Liu HJ Wang F Liang YY Hong JM Ma X Xu Z Shape controllable preparation of PbS crystals by a simple aqueous phase route Cryst Grow Des 2004 4 759 764 10.1021/cg034103f

[B20] Ye XD Duan YG Ding YC Research on the forming mechanism of micro/nano features during the cast molding process Nano-Micro Lett 2011 3 249 255

[B21] Bashouti M Lifshitz E PbS sub-micrometer structures with anisotropic shape: Ribbons, wires, octapods, and hollowed cubes Inorg Chem 2008 47 678 682 10.1021/ic700706a 18154328

[B22] Zhou GJ Lü MK Xiu ZL Wang SF Zhang HP Zhou YY Wang SM Controlled synthesis of high-quality PbS star-shaped dendrites, multipods, truncated nanocubes, and nanocubes and their shape evolution process J Phys Chem B 2006 110 6543 6548 10.1021/jp0549881 16570952

[B23] Zhang C Kang ZH Shen EH Wang EB Gao L Luo F Tian C Wang C Lan Y Li J Cao X Synthesis and evolution of PbS nanocrystals through a surfactant-assisted solvothermal route J Phys Chem B 2006 110 184 189 10.1021/jp053215+ 16471519

[B24] Kuang DB Xu AW Fang YP Liu HQ Frommen C Fenske D Surfactant-assisted growth of novel PbS dendritic nanostructures via facile hydrothermal process Adv Mater 2003 15 1747 1750 10.1002/adma.200304623

[B25] Wang N Cao X Guo L Yang S Wu Z Facile synthesis of PbS truncated octahedron crystals with high symmetry and their large-scale assembly into regular patterns by a simple solution route ACS Nano 2008 2 184 190 10.1021/nn7000855 19206617

[B26] Zhang ZH Lee SH Vittal JJ Chin WS A simple way to prepare PbS nanocrystals with morphology tuning at room temperature J Phys Chem B 2006 110 6649 6654 16570968 10.1021/jp057271m

[B27] Quan ZW Li CX Zhang XM Yang J Yang PP Zhang CM Lin J Polyol-mediated synthesis of PbS crystals: shape evolution and growth mechanism Cryst Grow Des 2008 8 2384 2392 10.1021/cg701236v

[B28] Chen M Xie Y Yao Z Qian Y Zhou G A novel solvent-directed shape control technique for preparation of rod-shaped PbS crystals Mater Res Bull 2002 37 247 253 10.1016/S0025-5408(01)00782-6

[B29] Xiang JH Cao HQ Wu QZ Zhang SC Zhang XR L-cysteine-assisted self-assembly of complex PbS structures Cryst Grow Des 2008 8 3935 3940 10.1021/cg7007842

[B30] Ma YR Qi LM Ma JM Cheng HM Hierarchical, star-shaped PbS crystals formed by a simple solution route Cryst Grow Des 2004 4 351 354 10.1021/cg034174e

[B31] Wang ZL Transmission electron microscopy of shape-controlled nanocrystals and their assemblies J Phys Chem B 2000 104 1153 1175 10.1021/jp993593c

