# Supplementary material for: Solvothermal synthesis, growth mechanism, and photoluminescence property of sub-micrometer PbS anisotropic structures
Source: Nanoscale Res Lett. 2012 Dec 6;7(1):668. doi: 10.1186/1556-276X-7-668 (PMC3552824; doi:10.1186/1556-276X-7-668)
Supplement: Additional file 1 — The structure synthesized with different SDBS concentrations was shown in Figure S1. The TG-DTA and element analyses of precursor were described in Figure S2 and Table S1. [file 1556-276X-7-668-S1.doc]

**Supplementary Materials for**

Solvothermal synthesis, growth mechanism and photoluminescence property of sub-micrometer PbS anisotropic structures

Yali Cao^1^, Pengfei Hu^2^, Dianzeng Jia^1^,***

*^1^Key Laboratory of Advanced Functional Materials of Autonomous Rregion, Key Laboratory of Clean Energy Material and Technology of Ministry of Education, Institute of Applied Chemistry, Xinjiang University, Urumqi, Xinjiang 830046, China*

*^2^Laboratory for Microstructures, Shanghai University, Shanghai 200444, China*

^*^Corresponding author. Tel: +86-991-8588883. Fax: +86-991-8588883. E-mail addresses: [jdz0991@gmail.com](mailto:jdz0991@gmail.com)


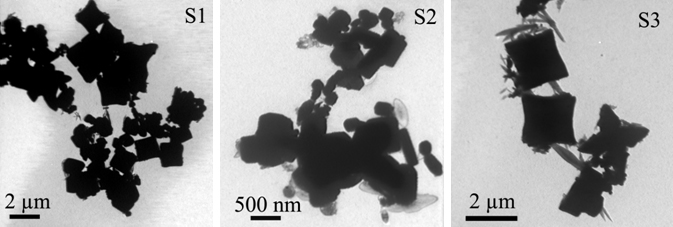


**Figure S1 TEM images of the samples synthesized at reaction temperature 180 °C with different mass of SDBS**. (S1) 0.8×10^-3^ M. **(S2)** 1.2×10^-3^ M. **(S3)** 7.2×10^-3^ M.


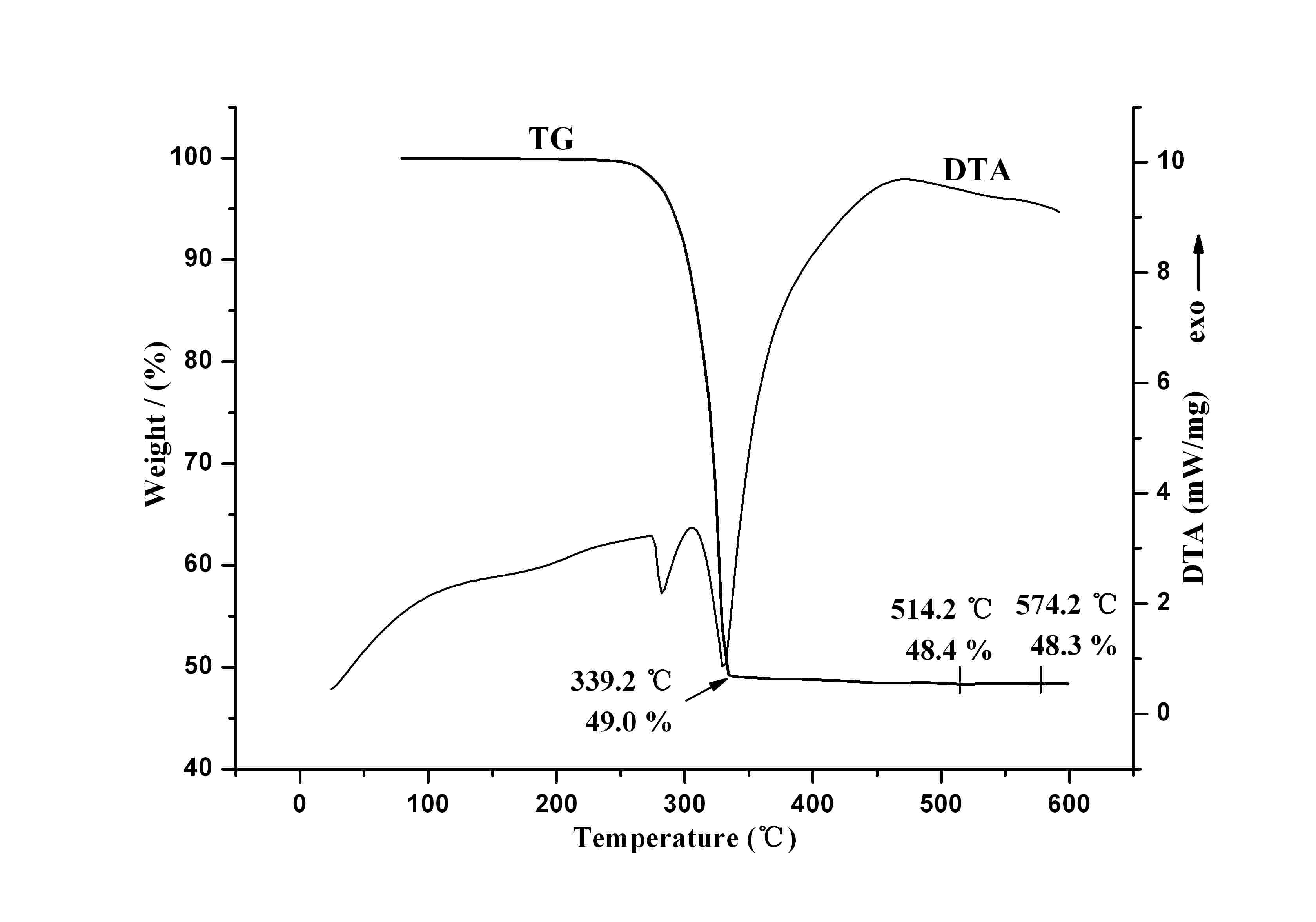


**Figure S2** **TG and DTA curves of the Pb(SNC_5_H_8_)_2_ precursor.**

The TG-DTA analysis for precursor: TG results indicated that decomposition of Pb(SNC_5_H_8_)_2_ precursor began at above 200 °C, giving 49.0% residue at 339.2 °C. It is not in good agreement with the calculated value for the PbS residue (47.9%). The weight loss slowly continue beyond 339.2 °C, for example, giving 48.4%, 48.3% residue at 514.2 °C, 574.2 °C respectively. We speculated that the as-abtained products at 339.2 °C probably are not 1:1 molar ratio of S/Pb, they has high molar ratio of S/Pb (> 1:1). So the weight residue is higher than the calculated value. Further investigations are currently underway to explore the underlying reason about the TG of precursor. The element analysis results confirm the composition of precursor.

**Table S1** **Element analysis for precursor (%)**

|  | C | H | N | S |
| --- | --- | --- | --- | --- |
| Found values  Calcaulated values | 24.4  24.0 | 3.0  3.2 | 5.4  5.6 | 25.3  25.7 |
